# Indications and visual outcomes of intrastromal corneal ring segment implantation in a large patient series

**DOI:** 10.6061/clinics/2017(06)07

**Published:** 2017-06

**Authors:** Taíse Tognon, Mauro Campos, João Paulo Wengrzynovski, Kleyton Arlindo Barella, Adriano Pasqualotti, Luiz Antônio de Brito Martins, Adriana dos Santos Forseto, Luciene Barbosa de Sousa

**Affiliations:** I Instituto Penido Burnier, Campinas, SP, BR; II Hospital Oftalmologico de Sorocaba, Sorocaba, SP, BR; III Universidade Federal de Sao Paulo, Sao Paulo, SP, BR; IV Hospital de Olhos do Parana, Curitiba, PR, BR; V Universidade de Passo Fundo, Passo Fundo, RS, BR; VI Universidade de Lisboa, Lisboa, Portugal; VII Banco de Olhos de Goias, Goiania, GO, BR

**Keywords:** Epidemiology, Ophthalmologic Surgical Procedure, Cornea, Ectasia, Brazil

## Abstract

**OBJECTIVES:**

To describe the indications for and visual outcomes of intrastromal corneal ring segment implantation.

**METHODS:**

A large retrospective case-series chart-review study was conducted using Sorocaba Ophthalmological Hospital medical records. This study included 1222 eyes (1196 patients) that were surgically treated between November 2009 and December 2012. The following preoperative data were collected: age, gender, type of medical care and funding source, surgical technique, best-corrected visual acuity, manifest sphere and cylinder refractive error, maximum and minimum central keratometry, and pachymetry measurements of the cornea at the thinnest point and at the ring channel. The postoperative best-corrected visual acuity and patient satisfaction were also determined. The cases were classified into six groups: four keratoconus groups (severe, advanced, moderate and mild), a pellucid marginal degeneration group and a post-graft irregular astigmatism group. This study was approved by the Brazilian Registry of Clinical Trials (UTN number 1111-1182-6181, TRIAL RBR-6S72RF).

**RESULTS:**

The age (mean±standard deviation) of the patients was 31.0±10.0 years. The most prevalent pathology was keratoconus (1147 eyes, 93.8%). A correlation was found between ectasia severity and medical assistance (*p*<0.001), and the most serious cases was treated by the Brazilian public health system. No complications were found in a total of 1155 surgeries, and after surgery, 959 patients were satisfied. Among the 164 dissatisfied patients, the majority failed to show improved best-corrected visual acuity.

**CONCLUSION:**

Patients in the public health system underwent surgical intervention for keratoconus later than those with private sources of funding. In the vast majority of operated cases, the patients reported improvements in vision.

## INTRODUCTION

Corneal deformities that lead to a loss of visual acuity have historically been a challenge for ophthalmologists. These deformities are primarily the result of corneal ectasias, such as pellucid marginal degeneration and keratoconus. Keratoconus is also the most common ectatic pathology, with a prevalence ranging from nine to 229 per 100,000 individuals across different populations 1,2.

Several techniques have been developed over the years to improve outcomes in these cases. Treatment can consist of spectacles, contact lenses, keratoplasty and, more recently, crosslinking and intracorneal implants 2,3.

Corneal ring implants were initially used to treat low myopia 4,5, but current applications include treatments for keratoconus, irregular astigmatism induced by penetrating keratoplasty, post-refractive surgery ectasia, post-radial keratotomy ectasia, pellucid marginal degeneration and post-traumatic corneal irregularities 6-8.

Corneal ring implantation aims to restore ectatic corneas by reducing corneal steepening and decreasing irregular astigmatism, thereby improving visual acuity 9. Many authors have emphasized the advantages of the implants, including their removable nature and their stability and security due to the lack of need for an intraocular procedure 6-11.

Recently, several surgical nomograms have been proposed for corneal ring implantation. These are mainly based on spherocylindrical error, the morphological and topographical characteristics of the corneal deformity, and aberrometric alterations 12.

With the development of femtosecond technology, corneal ring implantation has become safer. Surgery using this technology appears to result in fewer complications than does surgery based on mechanical (exclusively manual) techniques 6,13-15. Femtosecond-assisted implantation can even be combined with other procedures, such as crosslinking and refractive ablation 16,17.

Although research in this area has increased, there has been little emphasis on the corresponding epidemiological profiles of the evaluated patients. It has been shown that a proper understanding of these characteristics is crucial for promoting health.

This study aimed to present epidemiological data from patients undergoing corneal ring surgery in a tertiary hospital in Brazil and to delineate the indications for the procedures as well as the visual outcomes.

## MATERIALS AND METHODS

### Subjects

This large retrospective case-series study was based on data obtained at Sorocaba Ophthalmological Hospital (Sorocaba, São Paulo, Brazil), was developed in accordance with the principles of the Declaration of Helsinki and was approved by the Committee of Ethics and Research of Sorocaba Ophthalmological Hospital (number 101.552) and São Paulo Federal University (number 1.309.808).

We analyzed records from 1355 intrastromal corneal ring implantations (1238 patients) performed between November 2009 and December 2012. The inclusion criteria were as follows: patients who underwent corneal ring implantations at Sorocaba Ophthalmological Hospital during the period of interest using rings that were 5 mm in diameter and for whom surgery and medical records were available. The exclusion criteria were as follows: patients who did not previously undergo a complete preoperative ophthalmic evaluation, comprising corneal topography/tomography and pachymetry. In total, 1222 surgeries (1196 patients) were included in this study.

The following preoperative information was collected for all eligible individuals: age, gender, type of health/medical assistance (in the Brazilian public health system or via a health/medical organization or private payment), the surgical technique used to create the corneal ring tunnel (manual or femtosecond laser-assisted), best-corrected visual acuity (BCVA) (Snellen acuity converted to logMAR scale) 18, and manifest sphere and cylinder refractive error in diopters (D).

Additional data were obtained, such as maximum central keratometry (K, expressed in D and obtained from the central three millimeters (mm) of the corneal radius), minimum central K (expressed in D and obtained from the central 3 mm of the corneal radius), and corneal thickness (pachymetry) at the thinnest point and at the ring channel (both in micrometers, µm). The last four measurements were acquired using an Orbscan IIz^®^ system (Bausch & Lomb, Berlin, Germany).

The cases were classified into the following six groups according to their topographical characteristics and in consideration of their maximum central K and characteristics related to their astigmatism: mild keratoconus (up to 48D), moderate keratoconus (≥48D to 52D), advanced keratoconus (≥52D to 58D), severe keratoconus (≥58D), pellucid marginal degeneration and post-graft irregular astigmatism. These groups were adopted because current classification systems for ectasias vary widely and because classifications based exclusively on keratometry values were originally developed for topographic mapping, not tomographic mapping. Furthermore, classification systems tend to change over time, and the system adopted here avoids potentially outdated groupings.

Three months after the operation, information regarding visual acuity and satisfaction were collected. We classified patients as dissatisfied if contact lens or spectacle fitting was not possible according to medical records and/or the patient perceived that their eyesight had worsened after the procedure. Patients with intraoperative or postoperative complications were also considered dissatisfied.

### Intrastromal corneal ring surgery

A team with specific training in corneal surgery and at least one year of subspecialty in anterior segment disorders performed all the included surgeries at the previously mentioned hospital.

The procedures were performed under sterile conditions. The type of anesthesia (topical, topical with sedation or general anesthesia) was chosen according to the patient’s profile. A topical antibiotic (moxifloxacin 0.5%) and corticosteroid (prednisolone 1%) were administered four times per day for a course of seven days, and patients were instructed to wear therapeutic contact lenses for this period.

One of the following two techniques was used to create the ring tunnel: exclusively manual (mechanical) implantation or femtosecond laser-assisted implantation. In the mechanical method, a mark is first made using the Purkinje reflex as a guide 19. Then, a calibrated diamond knife is used to create a radial incision at 80% of the measured corneal thickness, which is determined via pachymetry 19. From the base of the incision, pocketing hooks are used to construct corneal pockets in the direction of the planned intrastromal corneal ring implant 19. These pockets are elongated using a glide-blade instrument 19. Next, one or two semicircular dissectors (clockwise and counterclockwise) are placed in the lamellar pocket and then steadily advanced using a rotational movement to create one or two semicircular tunnels into which the implants are inserted 19.

In femtosecond laser-assisted surgery, the Purkinje reflex is also marked as the central point 14. The femtosecond laser that was used in this study was an IntraLase^TM^ Laser FS150 (Abbott Medical Optics, Irvine, California, USA), and the implants were placed in a thinned region of the cornea that was 75% of its full thickness, as previously measured using pachymetry. The channel’s inner diameter was set to 5 mm, while its outer diameter was set to 5.9 mm. The energy used to create the channel was 1.10 µJ. The implantation of the intracorneal ring segments was performed immediately after the channel was created and before the bubbles disappeared, as they revealed the exact tunnel location 14.

Femtosecond laser-assisted ring implantation is the preferred surgical technique in this hospital, and its use is encouraged. However, its use depends on the availability of the laser and the cost of the surgery (the use of the femtosecond laser is not funded by the public system or by some health/medical organizations). In such situations, the cost must be covered by the patient or by teaching programs that are occasionally offered by the hospital.

All patients received Keraring^®^ (Mediphacos, Belo Horizonte, Brazil) intrastromal corneal ring implants. Keraring^®^ implants have a transverse triangular design, and they are available in a variety of crescent-shapes with a 5 mm radius of curvature (used in all patients in this study) with varying thicknesses (150 µm, 200 µm, 250 µm, 300 µm and 350 µm) and arc widths (90°, 120°, 160° and 210°). This allows multiple combinations to be used. For each case, the surgical plan was decided by the surgeon and a team of experts (at least five) according to the manufacturer’s nomogram.

### Statistical analysis

IBM SPSS22^®^ (SPSS Inc., Chicago, Illinois, USA) was used for descriptive and comparative analyses. Comparisons of two independent samples were analyzed using Student’s t-test for continuous variables and a Chi-square test for categorical variables. For multiple comparisons, ANOVA and Tukey’s test were used. We used Levene’s test to analyze homogeneity. A difference was considered significant at a *p* value <0.05.

### Ethics

This study was developed in accordance with the principles of the Declaration of Helsinki and was approved by the Committee of Ethics and Research of Sorocaba Ophthalmological Hospital (number 101.552) and São Paulo Federal University (number 1.309.808). It was also approved by the Brazilian Registry of Clinical Trials (UTN number 1111-1182-6181, TRIAL RBR-6S72RF).

## RESULTS

Of the 1222 included surgeries, we verified that 39 were performed in 2009 (November and December), 416 were performed in 2010, 395 were performed in 2011, and 372 were performed in 2012.

The demographic characteristics of the patients who underwent intrastromal corneal ring implantation at Sorocaba Ophthalmological Hospital between November 2009 and December 2012 are shown in Table 1. The age (mean±standard deviation (variation)) of these patients was 31.0±10.0 (range, 8-87) years. Separating the cases according to the type of health/medical care assistance used revealed that the majority were administered by the Brazilian public health system, which accounted for 858 cases (70.2% of the surgeries), followed by health/medical organizations (207 cases, 16.9%) and private coverage (157 cases, 12.8%). Intrastromal corneal ring implantation was performed to treat the following pathologies: severe keratoconus in 152 cases (12.5%), advanced keratoconus in 721 cases (59.1%), moderate keratoconus in 189 cases (15.5%), mild keratoconus in 85 cases (6.7%), pellucid marginal degeneration in 35 cases (2.9%), and post-graft irregular astigmatism in 40 cases (3.3%).

Table 2 shows the frequencies, distributions and comparisons between patients according to their source of health/medical care assistance. Women predominantly used the public health system, whereas men were more likely than women to use health/medical organizations or private payment (*p*<0.001). The mechanical technique was more frequently performed on patients who used the public system, while femtosecond laser-assisted surgery was more common for patients who used health/medical organizations or private coverage (*p*<0.001).

Severe keratoconus was less prevalent among patients using private coverage, whereas advanced keratoconus was more prevalent among patients using the Brazilian public health system. Moderate keratoconus was more prevalent among patients with private coverage, and mild keratoconus was more common among patients who used health/medical organizations or private payment (*p*<0.001). Significant differences were also found between patients using these types of health/medical assistance in the mean manifest preoperative cylinder refractive error (*p*=0.004), the mean maximum and minimum central K (*p*<0.001), and the mean thinner thickness pachymetry (*p*=0.013).

Table 3 shows the frequency and distribution of patients who underwent intrastromal corneal ring implantation according to the type of surgical technique used and the results of the comparisons between the two groups. In this analysis, a higher than average percentage of males were treated with femtosecond laser assistance, and a higher than average percentage of females were in the mechanically treated group (*p*=0.006). Significant differences were also reported in the mean maximum central K (*p*=0.018) and the mean thinner thickness pachymetry (*p*=0.009). Differences between the groups were also found regarding post-surgical satisfaction (*p*<0.001).

Table 4 compares the mean preoperative and postoperative BCVA by gender, type of surgical technique, type of medical assistance and type of corneal pathology in 959 patients who were satisfied postoperatively. In all the studied groups, visual acuity improved significantly (*p*≤0.003).

Table 5 shows the mean preoperative and postoperative BCVA of 164 patients who were dissatisfied postoperatively. This group included 67 patients who experienced surgical complications. The reported surgical complications consisted of the following: external environment or anterior chamber perforation, late (≥30 days) or early infection, late or early segment extrusion and malposition/movement of the intrastromal corneal ring segments after the procedure.

We found that in the majority of the patients in the dissatisfied group, the mean BCVA did not significantly improve after the procedure.

A total of 99 patients were excluded from this analysis because no postoperative assessment of satisfaction was included in their medical records.

Table 6 reports the number and frequency of patients in each category of medical assistance according to the visual impairment classification of the World Health Organization before and after intrastromal corneal ring implantation. Before the surgical procedure, the distribution of visual impairment was not homogeneous, and the majority of patients were in the moderate visual impairment group. After the procedure, the distribution became more homogeneous, and larger numbers of patients with improved vision were found in the normal vision and mild visual impairment groups.

Seven patients with severe and profound visual impairment (logMAR ≥1.0) were excluded from the analysis due to incomplete medical records, which made it difficult to perform a statistical analysis of this group.

## DISCUSSION

In this study, we evaluated a large cohort of patients to identify the indications for and outcomes of corneal ring implantation. This is one of the first studies to report these parameters and to correlate them with the social and economic aspects of individuals living in Brazil.

Keratoconus was the most commonly reported corneal pathology. The results described in this study are consistent with those reported in the literature and confirm that keratoconus is the most common ectatic pathology 1,2.

Krachmer et al. suggested that patients without central corneal scars who have mild to moderate disease and who cannot tolerate contact lenses are the best candidates for intrastromal corneal implantation 20.

Intracorneal rings were initially proposed to correct ametropia 21-23 and were thereafter successfully used to treat mild to moderate keratoconus 3,24,25. They are also currently used to treat severe keratoconus 25-28, other types of corneal ectasia 29 and irregular astigmatisms 13.

However, these implants are contraindicated in patients who present with the following conditions: keratoconus maximum K exceeding 70 D, corneal opacities involving the visual axis (including hydrops), or irregular corneal scars 20. Intracorneal rings are also contraindicated in atopic patients with chronic itching, local or systemic immunosuppression, or active ocular infection, recurrent erosion or corneal dystrophy 20.

In our study, a significant number of patients presented with severe and advanced keratoconus. In these cases, intrastromal ring implants were used to improve corneal topography, which consequently allowed the measurement of refractive errors and contact lens fit. These implants enabled more invasive procedures to be postponed or avoided altogether 14,19.

Recent studies have shown that the assessment of anterior segment characteristics, especially anterior corneal curvature and pachymetry, is important for monitoring and managing ectatic disease 30. In the current study, patients in the Brazilian public health system presented at surgery with a higher mean manifest cylinder refractive error, a higher maximum and minimum central K and a thinner corneal thickness pachymetry than patients with other forms of health assistance, indicating that patients in the public health system had more advanced ectatic disease. These findings are likely the result of the social and economic barriers these patients encounter when identifying a reference center for appropriate treatment in Brazil 31. In this country, social and economic conditions vary widely among different parts of the population, and not all patients have access to adequate treatment options for these pathologies 31.

A large proportion of the poorest part of the population uses the Brazilian public health system as their form of medical assistance. In the present study, patients with the most severe disease used the Brazilian public health system. This finding suggests that patients have difficulty accessing health programs in their cities, which delays diagnosis. Furthermore, after a diagnosis is made, access to appropriate treatment options and reference centers is deficient. In addition, ectatic diseases are multifactorial, and social and environmental conditions, including diet, exposure to pollution, and the location of a patient’s residence, can exacerbate the pathology.

These socioeconomic factors can also explain the differences that were observed between genders and type of surgical technique used among the groups. It is possible that males have access to more resources than females, facilitating their use of health/medical organizations or private sources for femtosecond laser-guided surgery 31.

Patients in the mechanically treated group presented higher mean maximum central K and mean thinner thickness pachymetry values. The majority of these patients were assisted through the Brazilian public health system, and these patients had more advanced disease.

This study was conducted in a tertiary reference cornea treatment hospital. We suggest that the individuals in the private health care group included patients who could not find appropriate treatment options in their region of origin and therefore paid for the implant surgery.

The surgical results show that satisfied patients exhibited improvements in mean BCVA. Our results are in accordance with those reported elsewhere 9,24,25,28. It is important to highlight that this was not a prospective study.

It should be noted that some patients were dissatisfied with the results of intrastromal corneal ring implantation. This dissatisfaction may have been the result of corneal aberrations, changes in asphericity, or complications arising during or after the procedure. While some researchers believe that asphericity is a marker of visual quality 9, it was not possible to obtain measurements of asphericity in the present study.

Some authors have argued that the exclusively manual surgical technique increases the risk of complications relative to the femtosecond laser-assisted technique because of the imprecision in the former’s implantation depth throughout the tunnel dimension 27,32. These authors have suggested that using a femtosecond laser makes the procedure safer (by creating a more uniform tunnel depth) and more comfortable for both the patient and the surgeon. They have also reported similar BCVA results using the laser-assisted technique to those obtained using the manual technique when experienced surgeons performed the operation 6. In our study, both techniques resulted in improved mean BCVA values in the group of satisfied patients. No improvement was observed in the dissatisfied patients after the manual procedure, as described earlier.

It is important to highlight that the mechanical group included a greater proportion of dissatisfied patients and that no significant differences were found according to the ectasia classification group. These findings reinforce that the exclusively manual technique may be associated with an increased rate of dissatisfaction.

The majority of patients who were dissatisfied did not achieve optimal results after corneal ring implantation. This result also might have been due to variations in corneal biomechanical properties, such as the corneal resistance factor and corneal hysteresis 32. Future studies should investigate whether the corneal resistance factor, corneal hysteresis/elasticity or in vivo measurements of corneal water content can predict the amount of corneal flattening and the outcome of intracorneal ring segments in corneal ectasias 32. Another point of concern is that visual satisfaction may be related to the visual demands associated with social activities and the patient’s profession.

It is necessary to emphasize that intrastromal corneal rings were implanted in some patients with a normal BCVA; in these situations, the surgery was an endeavor to improve visual quality and/or the tolerability of spectacles or contact lenses.

The results of the present study and previous studies show that corneal ring implantation is effective in improving visual acuity. Therefore, coverage of the financial cost of this procedure should be considered by the Brazilian public health system and all medical/health organizations. Intrastromal corneal ring implantation can improve visual acuity and limit vision loss, and it has advantages over other surgeries (e.g., corneal transplantation), such as reduced cost, lower risk of complications, and earlier rehabilitation of patients into society 7,13,33-35.

Although this study included a large case series, it has some limitations, including its retrospective nature, incomplete access to some medical records, the exclusion of patients with postoperative evaluations that were performed at other centers (closer to the patients’ residences) and the restriction of the target population to a single tertiary reference hospital. Nonetheless, we believe that this study offers a foundation for further research on vision and health with a focus on epidemiological aspects.

## AUTHOR CONTRIBUTIONS

Tognon T was responsible for the study design, literature review, data collection and execution of all research steps. Campos M was responsible for the study design, coordination and critical review. Wengrzynovski JP was responsible for the literature review, data collection and critical review. Barella KA was responsible for the literature review, revision of the manuscript and critical review. Pasqualotti A was responsible for the study design, statistical analyses and critical review. Martins LA and Forseto AS were responsible for the study design and critical review. Sousa LB was responsible for the study design, coordination and critical review.

## Figures and Tables

**Table 1:** Demographic characteristics of patients who underwent intrastromal corneal ring implantations at Sorocaba Ophthalmological Hospital between November 2009 and December 2012.

Demographic characteristics	Eyes (n=1222)
Age (years; mean±SD)	31.0 (±10.0)
Health care assistance	
Brazilian public health system [%]	858 [70.2%]
Health/medical organizations [%]	207 [16.9%]
Private coverage [%]	157 [12.8%]
Type of surgical technique	
Manual [%]	323 [26.4%]
Femtosecond laser-assisted [%]	899 [73.6%]
Type of corneal pathology	
Severe keratoconus [%]	152 [12.5%]
Advanced keratoconus [%]	721 [59.1%]
Moderate keratoconus [%]	189 [15.5%]
Mild keratoconus [%]	85 [6.7%]
Pellucid marginal degeneration [%]	35 [2.9%]
Post-graft irregular astigmatism [%]	40 [3.3%]
Mean best-corrected visual acuity (logMAR; mean±SD)	0.53 (±0.22)
Mean manifest sphere refractive error (diopters; mean±SD)	-5.36 (±10.12)
Mean manifest cylinder refractive error (diopters; mean±SD)	-4.78 (±2.23)
Mean maximum central K (diopters; mean±SD)	54.22 (±4.17)
Mean minimum central K (diopters; mean±SD)	47.92 (±4.08)
Mean thinner thickness pachymetry (µm; mean±SD)	424.70 (±60.89)
Mean thinner thickness pachymetry at the corneal ring channel (µm; mean±SD)	500.52 (±53.32)

n=number; SD=standard deviation; µm=micrometers; K=keratometry.

**Table 2:** Frequency, distribution and comparison of patients who underwent intrastromal corneal ring implantations at Sorocaba Ophthalmological Hospital between November 2009 and December 2012 according to the source of health/medical care assistance.

Eyes (frequency [%])	Brazilian public health system 858 [70.2%]	Health/medical organizations 207 [16.9%]	Private coverage 157 [12.8%]	*p-*value^1^
Age (years; mean±SD)	30.6 (±9.5)	31.9 (±10.6)	31.7 (±11.2)	0.171
Gender				
Male [%]	389 [63.7%]	**119 [19.5%]**↑	**103 [16.9%]**↑	**<0.001**
Female [%]	**469 [76.8%]**↑	88 [14.4%]	54 [8.8%]	
Type of surgical technique				
Mechanical [%]	**298 [92.2%]**↑	8 [2.5%]	17 [5.3%]	**<0.001**
Femtosecond laser-assisted [%]	560 [62.3%]	**199 [22.1%]**↑	**140 [15.6%]**↑	
Type of corneal pathology				
Severe keratoconus [%]	117 [77.0%]	19 [12.5%]	**16 [10.5%]↓**	**<0.001**
Advanced keratoconus [%]	**541 [75.0%]↑**	**80 [11.1%]↓**	100 [13.9%]	
Moderate keratoconus [%]	**109 [57.7%]↓**	26 [13.7%]	**54 [28.6%]↑**	
Mild keratoconus [%]	40 [47.0%]	**20 [23.5%]↑**	**25 [29.5%]↑**	
Pellucid marginal degeneration [%]	23 [65.7%]	4 [11.5%]	8 [22.8%]	
Post-graft irregular astigmatism [%]	28 [70.0%]	8 [20.0%]	4 [10.0%]	
Mean best-corrected visual acuity (logMAR; mean±SD)	0.52 (±0.23)	0.52 (±0.23)	0.55 (±0.23)	0.444
Mean manifest sphere refractive error (diopters; mean±SD)	-5.68 (±9.34)	-4.30 (±15.27)	-4.70 (±4.45)	0.183
Mean manifest cylinder refractive error (diopters; mean±SD)	-4.91 (±2.24)^a^	-4.43 (±2.15)^b^	-4.41 (±2.20)^b^	**0.004**
Mean maximum central K (diopters; mean±SD)	54.79 (±3.80)^a^	52.46 (±4.67)^b^	53.44 (±4.69)^c^	**<0.001**
Mean minimum central K (diopters; mean±SD)	48.30 (±3.99)^a^	46.77 (±4.14)^b^	47.56 (±4.21)^ab^	**<0.001**
Mean thinner thickness pachymetry (µm; mean±SD)	421.65 (±59.29)^a^	435.23 (±64.87)^b^	427.62 (±62.89)^ab^	**0.013**
Mean thinner thickness pachymetry at the corneal ring channel (µm; mean±SD)	499.74 (±51.63)	505.49 (±59.11)	498.26 (±54.45)	0.326

SD=standard deviation; µm=micrometers; K=keratometry.

1 Comparisions among the Brazilian public health system, health/medical organizations and private coverage groups were performed using ANOVA and Tukey’s test for continuous variables. Chi-square test was performed for the assessment of categorical variables, and an ANOVA was performed for multiple variables.

↑ Percentage that was statistically higher than the average.

↓ Percentage that was statistically lower than the average.

a,b,c These letters identify differences between groups calculated using Tukey’s test according to the Brazilian public health system, health/medical organizations and private coverage groups.

**Table 3:** Frequency, distribution and comparison of patients who underwent intrastromal corneal ring implantations at Sorocaba Ophthalmological Hospital according to the type of surgical technique between November 2009 and December 2012.

Eyes (frequency [%])	Mechanical 323 [26.4%]	Femtosecond laser-assisted 899 [73.6%]	*p-*value^1^
Age (years; mean±SD)	31.6 (±9.6)	30.8 (±10.1)	0.190
Gender			
Male [%]	**140 [22.9%]**↓	**471 [77.1%]**↑	**0.006**
Female [%]	**183 [29.9%]**↑	**428 [70.1%]**↓	
Type of corneal pathology			
Severe keratoconus [%]	45 [29.6%]	107 [70.4%]	0.327
Advanced keratoconus [%]	189 [26.2%]	532 [73.8%]	
Moderate keratoconus [%]	50 [26.5%]	139 [73.5%]	
Mild keratoconus [%]	23 [27.1%]	62 [72.9%]	
Pellucid marginal degeneration [%]	10 [28.9%]	25 [71.1%]	
Post-graft irregular astigmatism [%]	6 [15.0%]	34 [85.0%]	
Mean best-corrected visual acuity (logMAR; mean±SD)	0.53 (±0.22)	0.53 (±0.23)	0.969
Mean manifest sphere refractive error (diopters; mean±SD)	-5.99 (±4.81)	-5.12 (±11.52)	0.189
Mean manifest cylinder refractive error (diopters; mean±SD)	-4.84 (±2.10)	-4.75 (±2.28)	0.541
Mean maximum central K (diopters; mean±SD)	54.60 (±4.05)	54.05 (±4.21)	**0.018**
Mean minimum central K (diopters; mean±SD)	48.22 (±3.91)	47.84 (±4.14)	0.154
Mean thinner thickness pachymetry (µm; mean±SD)	417.12 (±58.95)	427.44 (±61.41)	**0.009**
Mean thinner thickness pachymetry at the corneal ring channel (µm; mean±SD)	502.32 (±53.45)	499.92 (±53.30)	0.489
Postoperative satisfaction*			
Satisfied (number, frequency [%])	234 [24.4%]	725 [75.6%]	**<0.001**
Dissatisfied (number, frequency [%])	80 [48.8%]	84 [51.2%]	

SD=standard deviation; µm=micrometers; K=keratometry.

1 Comparisons of types of surgical technique were performed using Student’s t-test for continuous variables and the Chi-square test for categorical variables.

↑ Percentage that was statistically higher than the average.

↓ Percentage that was statistically lower than the average.

* Information regarding patient satisfaction was obtained three months after surgery. Dissatisfied patient: no contact lenses or spectacles were fitted according to the medical records, the perceived BCVA was worse after the procedure, and/or intraoperative or postoperative complications occurred.

**Table 4:** Visual outcomes of 959 satisfied intrastromal corneal ring implantation patients at Sorocaba Ophthalmological Hospital according to gender, type of surgical technique, medical assistance and corneal pathology between November 2009 and December 2012.

Variable (valid number)	Mean BCVA*, preoperative (±SD)	Mean BCVA*, postoperative (±SD)	*p-*value^1^
Gender			
Male (476)	0.51 (±0.22)	0.30 (±0.22)	<0.001
Female (483)	0.53 (±0.22)	0.34 (±0.23)	<0.001
Type of surgical technique			
Mechanical (234)	0.52 (±0.22)	0.33 (±0.23)	<0.001
Femtosecond laser-assisted (725)	0.51 (±0.22)	0.32 (±0.23)	<0.001
Health care assistance			
Brazilian public health system (686)	0.51 (±0.22)	0.35 (±0.23)	<0.001
Health/medical organizations (156)	0.50 (±0.22)	0.28 (±0.25)	<0.001
Private coverage (117)	0.56 (±0.22)	0.22 (±0.18)	<0.001
Type of corneal pathology			
Severe keratoconus (113)	0.55 (±0.22)	0.37 (±0.24)	<0.001
Advanced keratoconus (579)	0.53 (±0.23)	0.34 (±0.22)	<0.001
Moderate keratoconus (147)	0.46 (±0.19)	0.25 (±0.22)	<0.001
Mild keratoconus (65)	0.50 (±0.24)	0.26 (±0.25)	<0.001
Pellucid marginal degeneration (24)	0.46 (±0.22)	0.24 (±0.22)	0.003
Post-graft irregular astigmatism (31)	0.51 (±0.21)	0.33 (±0.17)	<0.001

* BCVA=best-corrected visual acuity measured with spectacles on a logMAR scale; SD=standard deviation.

1 Comparisons were performed using paired Student’s t-tests.

**Table 5:** Visual outcomes of 164 dissatisfied intrastromal corneal ring implantation patients at Sorocaba Ophthalmological Hospital according to gender, type of surgical technique, medical assistance and corneal pathology between November 2009 and December 2012.

Variable (valid number)	Mean BCVA*, preoperative (±SD)	Mean BCVA*, postoperative (±SD)	*p-*value^1^
Gender			
Male (73)	0.60 (±0.27)	0.49 (±0.31)	0.019
Female (91)	0.56 (±0.25)	0.61 (±0.29)	0.180
Type of surgical technique			
Mechanical (80)	0.55 (±0.24)	0.51 (±0.29)	0.454
Femtosecond laser-assisted (84)	0.61 (±0.27)	0.60 (±0.32)	0.790
Health care assistance			
Brazilian public health system (145)	0.59 (±0.25)	0.58 (±0.30)	0.691
Health/medical organizations (10)	0.63 (±0.32)	0.62 (±0.24)	0.920
Private coverage (9)	0.41 (±0.26)	0.24 (±0.30)	0.276
Type of corneal pathology			
Severe keratoconus (30)	0.57 (±0.26)	0.63 (±0.32)	0.396
Advanced keratoconus (96)	0.61 (±0.25)	0.56 (±0.29)	0.131
Moderate keratoconus (29)	0.49 (±0.26)	0.48 (±0.30)	0.897
Mild keratoconus (2)	0.55 (±0.21)	0.60 (±0.14)	0.500
Pellucid marginal degeneration (4)	0.45 (±0.25)	0.52 (±0.36)	0.650
Post-graft irregular astigmatism (3)	0.76 (±0.40)	0.70 (±0.52)	0.802

* BCVA=best-corrected visual acuity measured with spectacles on a logMAR scale; SD=standard deviation.

Dissatisfied cases: no contact lenses or spectacles were fitted according to medical records, the perceived BCVA was worse after the procedure, and/or intraoperative or postoperative complications occurred.

1 Comparisons were performed using paired Student’s t-tests.

**Table 6:** Frequency of patients in each medical assistance category according to the visual impairment classification of the World Health Organization before and after intrastromal corneal ring implantation.

WHO* Classification for Visual Impairment^1^		Medical assistance groups (valid cases=1110)				*p*-value^2^
		Brazilian public health system	Health/medical organizations	Private coverage	Total	
BEFORE	Normal vision (logMAR<0.18)	40 (4.9%)	9 (5.5%)	7 (5.6%)	56 (5.0%)	0.494
	Mild vision loss (logMAR 0.18 to 0.48)	268 (32.6%)	60 (36.8%)	34 (27.0%)	362 (32.6%)	
	Moderate visual impairment (logMAR 0.54 to 0.90)	513 (62.5%)	94 (57.7%)	85 (67.5%)	692 (62.4%)	
AFTER	Normal vision (logMAR<0.18)	197 (24.0%)	73 (44.8%)	68 (54.0%)	338 (30.5%)	<0.001
	Mild vision loss (logMAR 0.18 to 0.48)	317 (38.6%)	46 (28.2%)	42 (33.3%)	405 (36.5%)	
	Moderate visual impairment (logMAR 0.54 to 0.90)	307 (37.4%)	44 (27.0%)	16 (12.7%)	367 (33.0%)	

* WHO=World Health Organization.

1 Best-corrected visual acuity.

2 Comparisons were performed using Chi-square t-tests.
